# Impact of Thrombocytopenia on Outcomes in Hospitalized Patients With Pneumonia, Chronic Obstructive Pulmonary Disease, and Asthma: A Nationwide Study (2016–2020)

**DOI:** 10.7759/cureus.80037

**Published:** 2025-03-04

**Authors:** Christian Siochi, Bolaji Durodola, Farishta Ali, Vaishvik K Patel, Chioma Nwachukwu, Ben Lerman, Aressa Canuto Miller, Stephen Jesmajian

**Affiliations:** 1 Internal Medicine, Montefiore New Rochelle Hospital, Albert Einstein College of Medicine, New Rochelle, USA; 2 School of Medicine, St. George’s University, True Blue, GRD

**Keywords:** asthma, copd, hematological, pneumonia, thrombocytopenia

## Abstract

Background*: *Pneumonia (PNA), chronic obstructive pulmonary disease (COPD), and asthma affect millions of patients every year, and thrombocytopenia is a common finding inside the hospital. In this analysis, the authors aim to investigate the impact of thrombocytopenia in patients admitted due to PNA, COPD, or asthma in terms of all-cause mortality, length of stay, resource utilization, and need for intubation.

Methods*:* This is an analysis of the National Inpatient Sample Database for the years 2016-2020. Patients admitted with a primary diagnosis of PNA, COPD, or asthma, with or without a secondary diagnosis of thrombocytopenia, were identified using the International Classification of Diseases, Tenth Revision, Clinical Modification (ICD-10-CM) codes. The primary outcome was all-cause mortality. Secondary outcomes were length of stay, resource utilization, and intubation during admission. Univariate analysis was done, and variables such as age, gender, race, Charlson comorbidity index, hospital location, size, region, teaching status, and insurance with p<0.2 were considered for adjustment on a subsequent multivariate analysis. STATA v.13 (StataCorp LLC, College Station, TX) was used for statistical analysis. Data were considered statistically significant if p-value <0.05.

Results: Among 2,993,792 adult patients admitted with a primary diagnosis of PNA, 148,260 (4.95%) had thrombocytopenia. Of 2,637,483 admitted due to COPD, 77,160 (2.92%) had thrombocytopenia. Of 491,990 admitted due to asthma, 6,300 (1.28%) had thrombocytopenia. Thrombocytopenia was associated with significantly increased in-hospital mortality across all three conditions (PNA: OR 2.31; COPD: OR 2.99; asthma: OR 7.26; all p<0.001), along with prolonged length of stay, higher resource utilization, and increased intubation rates. Strikingly, patients with asthma had an increased in-hospital mortality by 626% compared to patients without thrombocytopenia.

Conclusion: PNA, COPD, and asthma patients with concomitant thrombocytopenia experienced significant adverse in-hospital outcomes. Further investigation is warranted to determine whether interventions targeting thrombocytopenia can mitigate these negative consequences.

## Introduction

Thrombocytopenia, defined as a platelet count below 150,000/µL, is a frequent hematological finding in hospitalized patients, with a reportedly increased prevalence of 35-45% in the critically ill [1]. In addition to its well-established role in hemostasis, platelets are also becoming recognized for their role in regulating immune responses, coagulation, and inflammation [2]. These processes are all key in the pathophysiology of respiratory diseases, such as asthma, pneumonia (PNA), and chronic obstructive pulmonary disease (COPD). These are all highly prevalent conditions that have resulted in a significant increase in hospitalization rates over the past decade [3]. According to the World Health Organization (WHO), COPD and lower respiratory tract infections, including PNA, are among two of the top 10 leading causes of mortality worldwide [4]. Moreover, thrombocytopenia has been correlated with an increased risk of mortality, with a notable association in patients suffering from respiratory diseases [5].

While thrombocytopenia has been thoroughly studied in critically ill patients, less is known about how it specifically affects patients with long-term respiratory diseases, such as PNA, asthma, and COPD [6]. Research that links thrombocytopenia to higher rates of morbidity in COPD patients also highlights the need for more research on its impact on exacerbations and quality of life outcomes [7]. For example, some evidence suggests that thrombocytopenia has been linked to worse outcomes, such as increased mortality, intensive care unit (ICU) transfers, and the need for mechanical ventilation during COPD exacerbations [8]. These results are contradicted by other research, which indicates that thrombocytosis - an elevated platelet - during acute exacerbations of COPD (AECOPD) is also similarly associated with mortality [9]. In the same way, it is unknown how platelet count and mortality relate to stable COPD. According to one study, people with COPD who have either thrombocytopenia or thrombocytosis are at a higher risk of exacerbations and have worse clinical outcomes [10]. Similarly, some research has demonstrated that patients with PNA who develop thrombocytopenia also have increased rates of sepsis, ICU hospitalizations, and the need for invasive mechanical ventilation [11].

Thrombocytopenia has a significant impact on the treatment and prognosis of patients with PNA and other lung diseases. Low platelet counts complicate clinical care by increasing the risks of bleeding, delaying critical interventions, and worsening overall outcomes, such as prolonged hospital stays and increased mortality [11]. Early identification and treatment of thrombocytopenia in these patients is essential because prompt actions can minimize complications, maximize treatment plans, and possibly enhance the patient's prognosis and standard of care. A deeper understanding of the connection between thrombocytopenia and these respiratory disorders may result in improved risk assessment and more individualized treatment regimens, which would ultimately benefit these susceptible patients.

Study aim

This study investigates the impact of thrombocytopenia on hospitalization outcomes in patients admitted for COPD, PNA, or asthma exacerbations. It will specifically look into how thrombocytopenia influences important clinical outcomes such as all-cause mortality, length of hospital stay, overall hospital costs, intubation rates, and requirement for blood transfusions. By analyzing these variables, our study aims to shed light on the role that thrombocytopenia plays in the deteriorating prognosis of hospitalized respiratory patients and to offer guidance for more effective management strategies.

This article consolidates three poster presentations previously presented at the CHEST 2024 Annual Meeting (October 6-9, 2024).

## Materials and methods

Study design

This was a retrospective cohort study using discharge data from the National Inpatient Sample (NIS) and Healthcare Cost and Utilization Project (HCUP), Agency for Healthcare Research and Quality from 2016 to 2020.

Study inclusion criteria

Patients with the International Classification of Diseases, Tenth Revision, Clinical Modification (ICD-10-CM) of PNA, COPD exacerbation, or asthma, aged >18 years, with or without a secondary diagnosis of thrombocytopenia, were identified using International Classification of Disease, Tenth Edition, Procedure Coding System (ICD-10-PCS) and ICD-10-CM codes [12].

Ethical considerations

The data from the NIS-HCUP is publicly available, deidentified, and exempt from institutional review board approval. The need for informed consent was waived.

Outcome measures

The primary outcome of interest is all-cause in-hospital all-cause mortality, while secondary outcomes include length of stay, total hospital charges, and rate of endotracheal intubation.

Statistical analysis

This study used a confidence interval (CI) of 95% and a p-value <0.05 as statistically significant in its analysis. Continuous variables were examined through the calculation of means accompanied by standard deviations or medians along with interquartile ranges in the case of normally distributed and skewed data, respectively. Descriptive statistics incorporating frequencies and percentages were employed for the analysis of categorical variables. Patient and hospital-level baseline characteristics, and in-hospital outcomes, were compared between patients with a primary diagnosis of PNA, COPD, or asthma, aged >18 years, with or without a secondary diagnosis of thrombocytopenia using the Pearson χ2 test for categorical variables and the independent sample t-test for continuous variables. To calculate unadjusted and adjusted odds ratios for in-hospital clinical outcomes, univariate and multivariate logistic regression were used. First, a univariate analysis was used to identify patient and hospital-level baseline characteristics with a p-value <0.2. A multivariate analysis was then done to adjust for these significant baseline characteristics to account for potential confounding factors. All analyses were conducted using STATA v.13 (StataCorp LLC, College Station, TX).

## Results

Baseline patient characteristics, including race and Charlson comorbidity index, and hospital-level characteristics are shown in Table 1 for the PNA cohort, Table 2 for COPD, and Table 3 for asthma. In the United States, between the years 2016 and 2020, among 2,993,792 adult patients admitted with a primary diagnosis of PNA, 148,260 (4.95%) had thrombocytopenia, and 2,845,532 (95.04%) did not. Among 2,637,483 admitted due to COPD, 77,160 (2.92%) had thrombocytopenia, and 2,560,323 (97.07%) did not. Among 491,990 admitted due to asthma, 6,300 (1.28%) had thrombocytopenia, and 485,690 (98.72%) did not.

**Table 1:** Percentage of patients with a primary diagnosis of PNA, per baseline patient and hospital-level characteristics, with or without a secondary diagnosis of thrombocytopenia. To account for potential confounding factors, a multivariate regression model was later adjusted for patient and hospital-level baseline characteristics with a p-value of <0.2. PNA: Pneumonia

Characteristics		Without thrombocytopenia		With thrombocytopenia		Total patients with PNA		p-value
Baseline characteristics of PNA patients		N	%	N	%	N	%	
Sex	Male	1,325,448.806	46.58	86,064.93	58.05	1,411,572.928	47.15	<0.001
	Female	1,520,083.194	53.42	62,195.07	41.95	1,582,219.072	52.85	
Race	White	2,127,319.723	74.76	109,000.752	73.52	2,236,362.624	74.7	<0.001
	Black	352,845.968	12.4	17,064.726	11.51	369,733.312	12.35	
	Hispanic	224,512.4748	7.89	13,224.792	8.92	237,707.0848	7.94	
	Asian	55,772.4272	1.96	4210.584	2.84	60,175.2192	2.01	
	Native American	19,634.1708	0.69	1052.646	0.71	20,657.1648	0.69	
	Other	65,447.236	2.3	3706.5	2.5	69,156.5952	2.31	
Charlson comorbidity index	0	442,480.226	15.55	14,455.35	9.75	456,852.6592	15.26	<0.001
	1	711,667.5532	25.01	22,239	15	733,778.4192	24.51	
	2	553,171.4208	19.44	25,426.59	17.15	578,400.6144	19.32	
	> 3	1,139,351.013	40.04	8613.906	5.81	1,224,760.307	40.91	
Median income ($)	<49999	956,952.4116	33.63	45,189.648	30.48	1,002,022.182	33.47	<0.001
	50000-64999	805,854.6624	28.32	40,074.678	27.03	845,746.24	28.25	
	65000-85.999	622,886.9548	21.89	34,559.406	23.31	657,436.7232	21.96	
	>86000	459,837.9712	16.16	28,436.268	19.18	488,287.4752	16.31	
Insurance type	Medicare	1,989,880.528	69.93	107,859.15	72.75	2,097,750.054	70.07	<0.001
	Medicaid	310,732.0944	10.92	15,078.042	10.17	326,023.9488	10.89	
	Private insurance	457,276.9924	16.07	21,986.958	14.83	479,306.0992	16.01	
	Self Pay	87,357.8324	3.07	3350.676	2.26	90,711.8976	3.03	
Hospital region	Northeast	487,724.1848	17.14	25,426.59	17.15	513,135.9488	17.14	<0.001
	Midwest	697,439.8932	24.51	34,455.624	23.24	731,982.144	24.45	
	South	1,217,887.696	42.8	60,920.034	41.09	1,278,648.563	42.71	
	West	442,480.226	15.55	27,457.752	18.52	470,025.344	15.7	
Hospital bed size	Small	806,423.7688	28.34	35,760.312	24.12	842,153.6896	28.13	<0.001
	Medium	856,505.132	30.1	44,982.084	30.34	901,430.7712	30.11	
	Large	1,182,603.099	41.56	6,7532.43	45.55	1,250,207.539	41.76	
Hospital location	Rural	550,894.9952	19.36	19,659.276	13.26	570,616.7552	19.06	<0.001
	Urban	2,294,637.005	80.64	128,600.724	86.74	2,423,175.245	80.94	
Hospital teaching status	No	1,306,668.294	45.92	58,518.222	39.47	1,365,169.152	45.6	<0.001
	Yes	1,538,863.706	54.08	89,741.778	60.53	1,628,622.848	54.4	
Age		68.8		69.87		-		

**Table 2:** Percentage of patients with a primary diagnosis of COPD, per baseline patient and hospital-level characteristics, with or without a secondary diagnosis of thrombocytopenia. To account for potential confounding factors, a multivariate regression model was later adjusted for patient and hospital-level baseline characteristics with a p-value of <0.2. COPD: Chronic obstructive pulmonary disease

Characteristics		Without thrombocytopenia		With thrombocytopenia		Total patients with PNA		p-value
Baseline characteristics of COPD patients		N	%	N	%	N	%	
Sex	Male	1,077,895.983	42.1	41,805.288	54.18	1119611.534	42.45	<0.001
	Female	1,482,427.017	57.9	35,354.712	45.82	1,517,871.467	57.55	
Race	White	1,964,791.87	76.74	59,737.272	77.42	2,024,531.951	76.76	<0.001
	Black	369,454.6089	14.43	9799.32	12.7	379,270.0554	14.38	
	Hispanic	138,513.4743	5.41	4629.6	6	143,215.3269	5.43	
	Asian	28,419.5853	1.11	1211.412	1.57	29,539.8096	1.12	
	Native American	13,313.6796	0.52	408.948	0.53	13,714.9116	0.52	
	Other	45,829.7817	1.79	1381.164	1.79	47,210.9457	1.79	
Charlson comorbidity index	0	0	0	0	0	0	0	<0.001
	1	870,253.7877	33.99	14,444.352	18.72	884,875.5465	33.55	
	2	652,370.3004	25.48	17,044.644	22.09	669,656.9337	25.39	
	> 3	1,037,442.88	40.52	45,671.004	59.19	1,083,214.268	41.07	
Median income ($)	<49999	987,516.5811	38.57	27,376.368	35.48	1,014,903.458	38.48	<0.001
	50000-64999	722,523.1506	28.22	21,134.124	27.39	743,506.4577	28.19	
	65000-85.999	519,489.5367	20.29	16,782.3	21.75	536,200.2939	20.33	
	>86000	331,049.7639	12.93	11,874.924	15.39	343,136.5383	13.01	
Insurance type	Medicare	1,812,708.684	70.8	56,674.02	73.45	186,9447.95	70.88	<0.001
	Medicaid	382,000.1916	14.92	11,188.2	14.5	392,984.967	14.9	
	Private insurance	295,461.2742	11.54	7631.124	9.89	303,046.7967	11.49	
	Self Pay	70,152.8502	2.74	1666.656	2.16	71,739.5376	2.72	
Hospital region	Northeast	483,388.9824	18.88	13,487.568	17.48	496,901.7972	18.84	<0.001
	Midwest	614,221.4877	23.99	17,877.972	23.17	631,940.9268	23.96	
	South	1,131,150.701	44.18	33,634.044	43.59	1,164,976.241	44.17	
	West	331,561.8285	12.95	12152.7	15.75	343,664.0349	13.03	
Hospital bed size	Small	701,528.502	27.4	19,127.964	24.79	720,560.3556	27.32	<0.001
	Medium	790,115.6778	30.86	23,055.408	29.88	813,136.0089	30.83	
	Large	1,068,678.82	41.74	34,976.628	45.33	1,103,786.636	41.85	
Hospital location	Rural	462,138.3015	18.05	9729.876	12.61	471,845.7087	17.89	<0.001
	Urban	2,098,184.699	81.95	67,430.124	87.39	2,165,637.291	82.11	
Hospital teaching status	No	1,166,483.159	45.56	31,373.256	40.66	1,197,681.03	45.41	<0.001
	Yes	1,393,839.841	54.44	45,786.744	59.34	143,9801.97	54.59	
Age		68.12		69.52		-		

**Table 3:** Percentage of patients with a primary diagnosis of asthma, per baseline patient and hospital-level characteristics, with or without a secondary diagnosis of thrombocytopenia. To account for potential confounding factors, a multivariate regression model was later adjusted for patient and hospital-level baseline characteristics with a p-value of <0.2.

Characteristics		Without thrombocytopenia		With thrombocytopenia		Total patients with PNA		p-value
Baseline characteristics of asthma patients		N	%	N	%	N	%	
Sex	Male	132,010.542	27.18	2194.92	34.84	134,165.673	27.27	<0.001
	Female	353,679.458	72.82	4105.08	65.16	357,824.327	72.73	
Race	White	215,403.515	44.35	3020.85	47.95	218,394.361	44.39	<0.001
	Black	157,703.543	32.47	1624.14	25.78	159,306.362	32.38	
	Hispanic	78,827.487	16.23	1082.34	17.18	79,899.176	16.24	
	Asian	13,162.199	2.71	262.71	4.17	13,431.327	2.73	
	Native American	3254.123	0.67	66.78	1.06	3345.532	0.68	
	Other	17,339.133	3.57	242.55	3.85	17,613.242	3.58	
Charlson comorbidity index	0	0	0	0	0	0	0	<0.001
	1	294,910.968	60.72	223.02	3.54	297,112.761	60.39	
	2	105,249.023	21.67	1329.93	21.11	106,614.233	21.67	
	> 3	85,530.009	17.61	2739.87	43.49	88,263.006	17.94	
Median income ($)	<49999	181,405.215	37.35	2145.78	34.06	183,561.469	37.31	<0.001
	50000-64999	120,256.844	24.76	1496.88	23.76	121,767.525	24.75	
	65000-85.999	104,763.333	21.57	1277.64	20.28	106,023.845	21.55	
	>86000	79,264.608	16.32	137.97	2.19	80,637.161	16.39	
Insurance type	Medicare	165,086.031	33.99	3255.21	51.67	168,358.978	34.22	<0.001
	Medicaid	144,929.896	29.84	1517.04	24.08	146,465.423	29.77	
	Private insurance	131,282.007	27.03	1220.31	19.37	132,492.907	26.93	
	Self Pay	44,392.066	9.14	307.44	4.88	44,672.692	9.08	
Hospital region	Northeast	127,493.625	26.25	1755.18	27.86	129,245.773	26.27	<0.001
	Midwest	94,612.412	19.48	1280.16	20.32	95,938.05	19.5	
	South	182,328.026	37.54	2000.25	31.75	184,348.653	37.47	
	West	81,255.937	16.73	1265.04	20.08	82,506.723	16.77	
Hospital bed size	Small	119,771.154	24.66	1525.23	24.21	12,1275.535	24.65	0.291
	Medium	155,177.955	31.95	1905.12	30.24	157,092.407	31.93	
	Large	210,740.891	43.39	2870.28	45.56	213,622.058	43.42	
Hospital location	Rural	39,243.752	8.08	401.94	6.38	39,654.394	8.06	0.0421
	Urban	446,446.248	91.92	5898.06	93.62	452,335.606	91.94	
Hospital teaching status	No	151,389.573	31.17	1733.13	27.51	153,156.487	31.13	0.0116
	Yes	334,300.427	68.83	4566.87	72.49	338,833.513	68.87	
Age		50.69		59.93		-		

The PNA population with thrombocytopenia had a mean age of 69.87 years, while those without thrombocytopenia had a mean age of 68.8 years. The COPD population with thrombocytopenia had a mean age of 69.52 years, while those without thrombocytopenia had a mean age of 68.12 years. The asthma population with thrombocytopenia had a mean age of 59.93 years, while those without thrombocytopenia had a mean age of 50.69 years.

The majority of patients included in this study overall were white and female. However, among the PNA population, the majority of patients with thrombocytopenia were male (86,065 (58.05%)) (Table 1). Among the COPD population, the majority of patients with thrombocytopenia were male as well (41,805 (54.18%)) (Table 2). Among the asthma population, the majority of patients with thrombocytopenia were female (4,105 (65.16%) (Table 3).

Primary outcomes

Adjusted outcomes showed that patients with a secondary diagnosis of thrombocytopenia admitted due to PNA, COPD, or asthma had an increased in-patient all-cause mortality risk of 122% (OR 1.22; 95% (CI 1.09-1.37; p=0.001)), 139% (OR 1.39; 95% (CI 1.13-1.71; p=0.002)), and 111% (OR 1.11; 95% (CI 4.33-84.45; p<0.001)), respectively (Table 4).

**Table 4:** Adjusted odds ratio for in-hospital all-cause mortality outcome of patients admitted for PNA, COPD or asthma, with secondary diagnosis of thrombocytopenia. This study used a confidence interval of 95% and a p-value <0.05 as statistically significant in its analysis. PNA: Pneumonia; COPD: Chronic obstructive pulmonary disease

In-patient mortality	Odds ratio	Std error	t	p value	95% Confidence interval	
PNA	1.96	0.06	21.03	<0.001	1.84	2.08
COPD	2.31	0.13	14.71	<0.001	2.07	2.59
Asthma	4.78	1.19	6.26	<0.001	2.93	7.8

Secondary outcomes

Adjusted outcomes showed that patients admitted for PNA, COPD, or asthma with a secondary diagnosis of thrombocytopenia spent more days in the hospital during their admission and spent more dollars on resource utilization (Table 5).

**Table 5:** Adjusted regression coefficient for length of stay and total hospital charges outcome of patients admitted for PNA, COPD, or asthma, with secondary diagnosis of thrombocytopenia. This study used a confidence interval of 95% and a p-value <0.05 as statistically significant in its analysis. PNA: Pneumonia; COPD: Chronic obstructive pulmonary disease

Length of stay	Coefficient	Std Error	t	p-value	95 Interval	
PNA	1.19	0.04	28.35	<0.001	1.11	1.28
COPD	1.46	0.05	27.23	<0.001	1.35	1.56
Asthma	1.53	0.2	7.71	<0.001	1.14	1.92
Total hospital charges	Coefficient	Std Error	t	p-value	95 Interval	
PNA	15,024.42	631.51	23.79	<0.001	13,786.6	16,262.24
COPD	16,626.25	767.99	21.65	<0.001	15,120.92	18,131.58
Asthma	18,814.76	2995.61	6.28	<0.001	12,943.1	24,686.41

Adjusted outcomes also showed that patients admitted for PNA, COPD, or asthma with a secondary diagnosis of thrombocytopenia had a higher risk of intubation during their hospital stay (Table 6).

**Table 6:** Adjusted odds ratio for endotracheal intubation outcome of patients admitted for PNA, COPD, or asthma, with a secondary diagnosis of thrombocytopenia. This study used a confidence interval of 95% and a p-value <0.05 as statistically significant in its analysis. PNA: Pneumonia; COPD: Chronic obstructive pulmonary disease

Endotracheal intubation	Odds Ratio	Std Error	t	p-value	95 Interval	
PNA	2.17	0.07	22.46	<0.001	2.03	2.32
COPD	3.08	0.13	27.4	<0.001	2.85	3.34
Asthma	4.09	0.58	9.89	<0.001	3.1	5.41

## Discussion

In this retrospective study of patients admitted for COPD exacerbation, asthma, and PNA, those with thrombocytopenia experienced significantly worse outcomes, including higher all-cause mortality risk, longer hospital stays, increased likelihood of intubation, and higher costs. This study paves the way for examining the possibility of mitigating adverse pulmonary outcomes by exploring interventions aimed at treating thrombocytopenia. To our knowledge, only a few studies have evaluated the relationship between thrombocytopenia and adverse outcomes in these patient groups. In the present study, thrombocytopenia was observed in 77,160 (2.9%) of COPD patients and 6,300 (1.2%) of asthmatic patients. Additionally, 148,260 (4.9%) of PNA patients had thrombocytopenia, and this is consistent with the study carried out by Mirsaeidi et al., which showed that 5% of community-acquired PNA presented with thrombocytopenia [13].

In a study showing mortality in COPD patients, the mortality rate in the thrombocytopenia group was 86.4%, and a significant difference was observed when compared to those who did not develop thrombocytopenia. They also emphasized that a 10% decrease in platelet count could be considered an independent predictor of ICU mortality [14]. Another study showed that the mortality rate is high (61.5%) when thrombocytopenia occurs in acute exacerbation of COPD patients [15]. Similar findings were reported in a multicenter retrospective study involving 822 patients admitted to the ICU for severe community-acquired PNA, where severe thrombocytopenia (<50,000 cells/μL) was identified as an independent predictor of mortality [16]. Prolonged thrombocytopenia and the absence of a relative increase in platelet count (defined as a 25% rise above the admission value) were found to be linked to higher mortality in critically ill patients [17]. A study showed that community-acquired PNA patients with thrombocytopenia significantly needed mechanical ventilation either invasive or non-invasive, when compared to community-acquired PNA patients both with thrombocytosis or with normal platelet count [12].

Possible explanations for the association between thrombocytopenia and poor clinical outcomes include the likelihood that thrombocytopenia may act as a marker of bacterial infection and sepsis, which can result in higher morbidity in patients with COPD exacerbation [8]. Sepsis is a leading cause of thrombocytopenia in critically ill patients [18]. Sepsis in turn is a leading cause of COPD exacerbation. Additionally, platelets have immune-modulating functions, releasing chemokines and cytokines and interacting with other platelets, monocytes, and neutrophils [19].

Furthermore, potential explanations for the link between thrombocytopenia and poor outcomes in PNA include its association with disseminated intravascular coagulation and severe sepsis [20]. Community-acquired PNA is a leading cause of severe sepsis and septic shock, accounting for up to 45% of hospital admissions related to these conditions [20]. Various mechanisms contribute to the development of thrombocytopenia in patients with sepsis. During sepsis, platelets are thought to become activated and adhere to the endothelium, resulting in their sequestration and destruction. Additionally, immune-mediated factors, such as nonspecific platelet-associated antibodies and cytokine-driven hemophagocytosis of platelets, can play a role in causing sepsis-induced thrombocytopenia [21].

Interestingly, there have been conflicting results regarding the impact of platelet count on clinical outcomes in COPD and asthma patients. Some reports have demonstrated that a prothrombotic state exists in COPD, whereas others described an increase in platelet activation in COPD patients. It has been reported that elevated platelet arginase activity is usually observed in COPD patients, and this is due to alterations in nitric oxide metabolism [22]. A study also showed that soluble P-selectin, which is a marker of platelet hyperactivity, was higher in COPD patients [23].

Similar to the above, the study by Ashitani et al. showed elevated levels of a platelet activation marker (beta-thromboglobulin) and coagulation-fibrinolysis markers (fibrinopeptide A, thrombin-antithrombin III complex, and tissue plasminogen activator-plasminogen activator inhibitor) in 40 COPD patients compared to a control group [24]. They deduced that this clotting factor serves as an independent predictor of acute exacerbations. During COPD exacerbation, there is a pronounced disruption in hemostatic balance due to increased platelet aggregation, triggered by acute gas exchange disturbances and hypoxemia. This heightened platelet activity directly damages lung vessels and promotes the release of mediators [25,26].

There is a paucity of studies to compare our results of thrombocytopenia and asthma adverse outcomes. Some studies have shown that, in patients not receiving inhaled corticosteroid treatment, elevated platelet brain-derived neurotrophic factor concentrations are associated with airflow limitation and airway hyperresponsiveness [27].

Regarding the duration of hospital stays and the overall costs associated with prolonged hospitalization, it is crucial to identify patients with a poor prognosis and high mortality risk early on and implement suitable treatments. Utilizing cost-effective markers, such as complete blood counts, which can be quickly assessed in any facility for predicting mortality and prognosis, offers both financial and time-saving benefits [28].

Study limitations

Our study has certain limitations. The National Inpatient Sample is an archive of hospital administrative data, but it lacks the detailed clinical information available in electronic medical records. The identification of clinical conditions and procedures relies on the accuracy of diagnosis and procedure codes provided by hospitals, which are prone to errors. Additionally, our study was a retrospective analysis of previously collected data. Because the data rely primarily on the review of charts that were not originally created for research work, some of the information may be missing, and the unavailability of information on confounders leads to bias. Another significant concern is the lack of data on platelet count fluctuations during hospital stays. It is said that changes in platelet count during the ICU stay are more predictive of outcomes than a single platelet count measured at ICU admission [17]. This parameter is, however, not included in our database. Additionally, biomarkers were not analyzed, and causative organisms were not examined to investigate their potential relationship with platelet count, although previous studies have shown no significant statistical difference. Despite the limitations mentioned above, the large sample size of this study significantly enhances the statistical power of its results.

## Conclusions

Thrombocytopenia was linked to poorer pulmonary outcomes and identified as a risk factor for increased mortality, extended hospital stays, higher intubation rates, and elevated healthcare costs in patients admitted for COPD exacerbations, asthma, and PNA. This study underscores the importance of assessing thrombocytopenia as an inflammatory marker and prognostic indicator in these respiratory conditions. Prospective cohort studies are therefore needed to further explore the impact of thrombocytopenia on asthma, COPD exacerbations, and PNA. In addition, further research is required to elucidate the specific role of platelets in the pathophysiology of these conditions.
